# 

**Published:** 1903-05-15

**Authors:** 


					﻿the
DENTAL REGISTER
EDITED BY
NELVILLE S. HOFF, D. D. S.,
ANN ARBOR, MICH.
Vol. LVII.	MAY 15• i<)o3.	5• >
PRICE, ONE DOLLAR A YEAR IN ADVANCE.
PUBLISHED MONTHLY BY
SAMUEL A. CROCKER & CO.,
THE OHIO DENTAL AND SURGICAL DEPOT.
35 to 31) 'W'. “tlx JSt., Cincinnati, O.
Entered at the Postoffice at Cincinnati, 0., as second-class mail matter.
				

